# Can guidelines rein in oxygen use? A retrospective cross-sectional study using routinely collected data

**DOI:** 10.1093/intqhc/mzad073

**Published:** 2023-09-20

**Authors:** Usman Talat, Kelly A Schmidtke, Saval Khanal, Alice M Turner, Ivo Vlaev

**Affiliations:** Alliance Manchester Business School, University of Manchester, M15 6PB, United Kingdom; University of Health Sciences and Pharmacy in St Louis, MO 63110, United States; Warwick Medical School, University of Warwick, Coventry CV4 7AL, United Kingdom; University of East Anglia, Norwich, Norfolk NR4 7TJ, United Kingdom; Institute of Applied Health Research, University of Birmingham, B15 2TT, United Kingdom; Warwick Business School, University of Warwick, Coventry CV4 7AL, United Kingdom

**Keywords:** health policy, guidelines, emergency care, intensive care, training/education, oxygen

## Abstract

Oxygen is one of the most commonly used emergency therapies. Like other therapies, oxygen can cause harm if used inappropriately. During the COVID-19 pandemic, guidelines were released to optimize oxygen and medication use. In the current study, we examine whether oxygen and medication use during the first wave of the COVID-19 pandemic was in concordance with new guidelines.

A retrospective cross-sectional study was conducted using routinely collected data from University of Birmingham NHS Foundation Trust in England. Patients were admitted between April 2020 and September 2020, were over the age of 18 years, and had a confirmed diagnosis of COVID-19. To assess adherence to the oxygen guidelines (i.e. SpO_2_ adherence), the percentage of times oxygen therapy was administered within, over, and under guideline specifications were calculated for patients overall, and then for patients with and without chronic obstructive pulmonary disease (COPD)/pulmonary disease separately. Next, two multinomial regression analyses were conducted to assess whether clinical processes, pre-admission diagnoses, and other demographic factors were related to oxygen use. Analysis 1 included patients not diagnosed with COPD/pulmonary disease. Analysis 2 included patients diagnosed with COPD/pulmonary disease. Results are reported as tallies, percentages, and odds ratios with 95% confidence intervals. To assess adherence to a new medication guideline, the percentage of patients administered oxygen and dexamethasone was calculated for those admitted after 25 June 2020.

The overall number of patients included in our SpO_2_ adherence analyses was 8751 (female = 4168). Oxygen was used within guideline specifications less than half the time, i.e. 41.6% (*n* = 3638/8751); non-adherence involving under-administering (3.5%, *n* = 304/8751) was markedly lower than over-administering (55.0%, *n* = 4809/8751). Adherence was higher for patients without COPD (43.7%, *n* = 3383/7741) than with COPD (25.2%, *n* = 255/1010). Under-administering was low across groups (non-COPD 3.5%, *n* = 274/7741 and COPD 2.9%, *n* = 30/1010). Over-administering was markedly lower for non-COPD (52.3%, *n* = 4084/7741) than COPD (71.8%, *n* = 725/1010) patients. Diagnoses associated with over-administering varied across the groups. Regarding the dexamethasone guidelines, of the 6397 patients admitted after the 24th of June, only 12.6% (*n* = 805) received dexamethasone.

Suboptimal use of oxygen and medication were common during the first wave of the COVID-19 pandemic. As found in previous studies, over-administering was more common than under-administering. The new guidelines issued during the COVID-19 pandemic were not by themselves sufficient to optimize oxygen use. Behavioural strategies are explored which may help policymakers optimize oxygen use.

## Introduction

Guidelines are recommended standards for clinical care that can promote quality and safety. Guidelines are produced internationally (e.g. by the World Health Organization), nationally, and locally to support care generally and within specialty areas. For practitioners, keeping up to date with evolving guidelines can be challenging. How often guidelines should be revised is debated, as are the necessary and sufficient conditions that should give rise to changes. Generally, guidelines are updated every three to five years, but significant medical events could trigger faster changes [1]. The COVID-19 pandemic was a significant global event that triggered an avalanche of changes. The current paper describes new guidelines released around oxygen and medication use during the first wave of the COVID-19 pandemic in England and whether guideline implementation was successful.

Oxygen is one of the most commonly used emergency therapies. Like other therapies, oxygen can be under- or over-used. While the harms caused by underuse are intuitive, i.e. suffocation, the harms caused by overuse may be overlooked [2]. In 2019, JAMA published a structured review on medical overuse, which included a systematic review and meta-analysis of 25 randomized clinical trials including 16 037 acutely ill adults. This review found that, compared to more conservative use, overuse of oxygen therapy increased in-hospital mortality (relative risk, 1.21; 95% CI, 1.03–1.43) and 30-day mortality (relative risk, 1.14; 95% CI, 1.01–1.28) [3]. In low-resource settings, oxygen may be overused where workers do not have the equipment necessary to measure the amount of oxygen used [4]. However, even in high-resource settings overuse persists. For example, in the United Kingdom, a 2015 audit of emergency oxygen therapy found that of the 14% of hospital patients given oxygen only 58% had valid prescriptions [5]. During the COVID-19 pandemic, oxygen overuse put excessive demands on hospital medical gas pipelines, resulting in surgeries being cancelled, patients being diverted to different hospitals, and a need to reconfigure ward space [6]. While some patients were well served by oxygen therapy, questions remain around whether use was optimal overall and whether guidelines are a sufficient mechanism to facilitate better oxygen-related decisions.

Early in the COVID-19 pandemic, two new guidelines were released to optimize oxygen use. The first was issued in April 2020 by the British Thoracic Society. This guideline recommended that health workers decrease the target saturation of peripheral oxygen (SpO_2_) levels measured by pulse oximetry from 94–98% to 92–96% for adult patients without chronic obstructive pulmonary disease (COPD) and associated conditions [7, 8]. This decrease matches guidelines set then by the Thoracic Society of Australia and New Zealand and the United States National Institute of Health. For patients with COPD/pulmonary disease, the recommendation remained lower, i.e. 88–92%, as these patients are more likely to experience adverse events at higher levels [9]. The second guideline was issued on 25 June 2020, after dexamethasone emerged as a therapeutic candidate to reduce in-patient mortality [10, 11]. Here, the National Institute of Health’s COVID-19 Treatment Guidelines Panel recommended administering dexamethasone for patients with COVID-19 on mechanical ventilation or who require supplemental oxygen [12].

The rapidly changing guidelines occurring early in the COVID-19 pandemic rendered implementation challenging. The objectives of the current study were to assess whether oxygen use adhered to the newly released SpO_2_ guidelines during the first wave of the COVID-19 pandemic in one NHS acute care Trust for patients overall, and for those with and without COPD/pulmonary disease separately. Adherence to the newly released dexamethasone guideline is also described. The discussion reviews these findings and puts forth behavioural strategies to bring oxygen use within guideline specifications.

## Methods

A retrospective cross-sectional study was conducted. Approvals to use the data were granted by University Hospital Birmingham NHS Foundation Trust’s clinical governance department and ethical approval for the study was granted by the University of Warwick’s Humanities & Social Sciences Research Ethics Committee (ID: HSSREC 197/19-20). Our results are reported in concordance with the STROBE statement [13]. The Trust is located in one of England’s most ethnically diverse areas and treats nearly 2.2 million patients annually across its three acute care hospitals. Oxygen saturations are recorded after first titrating to the point where the maximum amount of oxygen is delivered to meet the patient’s SpO_2_ goal, after which those readings are entered into the record. In the 12 months prior to the COVID-19 pandemic, a quality improvement project was undertaken in which staff were trained to deliver and record stable post titration levels consistently. By the end of this process, saturation levels were being reached and reliably recorded within 5 minutes [14]. In addition to the SpO_2_, health workers record the flow rates applied to achieve post-treatment saturation levels. The analysis was conducted using routinely collected data stored in the hospital’s electronic records during the first wave of COVID-19, from April 2020 to September 2020 inclusive.

### Participants

Records were restricted to patients who were admitted to the hospital, over the age of 18 years, and diagnosed with COVID-19 as confirmed by polymerase chain reaction testing. According to intention-to-treat principles for our regression analyses, only initial patient records were retained for our analysis, i.e. each record indicates a unique patient. A large number of multiple records were expected, as the hospital records each treatment as a unique event and patients may experience several oxygen events over the course of an in-patient visit. Records from unique patients with missing data were excluded from the final analyses, e.g. missing oxygen flow rate. Lastly, we excluded patients marked as having low adherence but receiving a flow rate of 15 litres per minute as these patients had likely reached maximum ward-level care. The numbers of multiple records and patients removed are described in the results section.

### Variables

Data were retrieved to capture factors related to clinical procedures, pre-admission diagnoses, and demographics. The clinical procedures included oxygen flow rates, whether dexamethasone was prescribed, the number of other therapies prescribed, and post-treatment oxygen saturation levels (SpO_2_). The oxygen flow rates were measured by oxygen flow metres and could range from 1 to 15 litres per minute. SpO_2_ levels were measured by pulse oximeters and could range from 0% to 100%.

Several pre-admission diagnoses were included in the analyses, as these may influence compliance. For example, patients diagnosed with a cerebrovascular accident or chronic heart failure may appear more breathless (thus triggering overuse). As with COPD, noting which diagnoses are associated with over-administering may inform training to bring oxygen use within guideline specifications. The pre-admission diagnoses included acute myocardial infarction, cancer, cerebrovascular accident, chronic heart failure, connective tissue disorder, chronic obstructive pulmonary disease and pulmonary disease, dementia, diabetes with induced complications, human immunodeficiency virus (HIV), liver disease, paraplegia, peptic ulcer, peripheral vascular disease, and renal disease. Demographic measurements included age and sex at birth.

### Bias

To reduce the influence of confounding factors, the measures obtained and ultimately included in our analyses were selected through consultation with a respiratory specialist, hospital administrators, and hospital pharmacists.

### Study size

The sample size for our analyses was not predetermined. Rather, all eligible records were considered, i.e. opportunistic sampling.

### Statistical analyses

Analyses were conducted using SPSS version 21. Descriptive statistics were calculated to describe the demographics for patients with and without a pre-admission diagnosis of COPD/pulmonary disease. To assess adherence to the new dexamethasone guideline, the percentage of patients administered oxygen and dexamethasone was calculated for those admitted after 25 June 2020. To assess adherence to the new SpO_2_ use guidelines, the percentage of times oxygen therapy was administered within, over, and under guideline specifications were calculated for patients overall, and then for patients with and without COPD/pulmonary disease separately.

Next, two subgroup multinomial regression analyses were conducted (Enter method). The first included patients not diagnosed with COPD/pulmonary disease. The second included patients diagnosed with COPD/pulmonary disease. The dependent variable for each analysis was assessed at three levels: adhering (which was always the reference group), over-administering, and under-administering. For the first analysis, post-treatment SpO_2_ levels for adhering were 92%–96%, over-administering were >96%, and under-administering were <92%. For the second analysis, including patients with COPD/pulmonary disease, adherence levels were reduced to 88%–92%; over- and under-administering levels were respectively adjusted.

The predictor variables included clinical procedures, pre-admission diagnoses, and demographics. The clinical procedures included oxygen flow rate, the number of other medication therapies prescribed, and whether dexamethasone was prescribed (yes/no). The pre-admission diagnoses were entered as dichotomous variables (i.e. present vs absent), including cancer, peptic ulcer, renal disease, connective tissue disorder, chronic heart failure, HIV, cerebrovascular accident, liver disease, diabetes, and dementia. The same demographics were entered into both analyses, including sex as a dichotomous variable and age as a continuous variable. As the analysis was run, multicollinearity was a concern for liver disease in the COPD analysis for the under-administered comparison, and it was removed by SPSS for that analysis only, and no other concerns remained (all tolerances above 0.10). The statistical significance of each factor was assessed using a 0.05 *P*-value. Statistical outputs are reported using percentages, tallies, and odds ratios with 95% confidence intervals.

## Results

**Figure 1:**
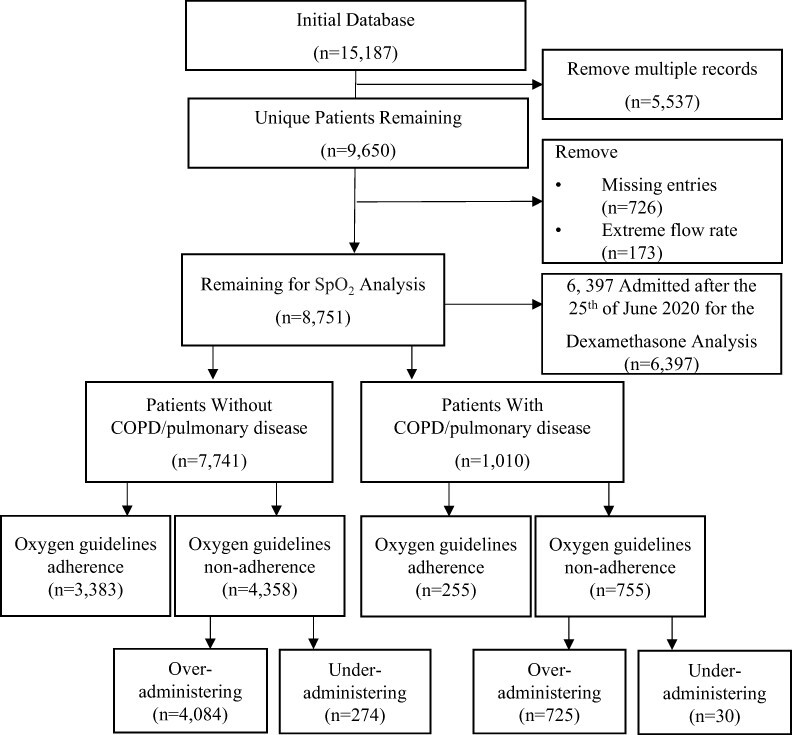
Prisma diagram conveying patients retained in the final analyses by COPD/pulmonary disease diagnosis.

From the initial data set of 15 187 records, 5537 repeated records were removed such that each record represents a unique patient. Of the remaining 9650 unique records, an additional 726 patients (7.4%) were removed with missing data. The most common missing data was oxygen flow rate (*n* = 718). Lastly, 173 patients with low adherence levels receiving a 15 l flow rate were removed. The final data set contained 8751 unique patients (4168 female), of which 7741 were without COPD/pulmonary disease and 1010 were with COPD/pulmonary disease. Figure 1 presents a flow chart describing patients in the dexamethasone and SpO_2_ analyses. Table 1 presents the patient characteristics of those with and without COPD/pulmonary disease. Patients without COPD/pulmonary disease tended to be younger (Mean = 62.50 years old, SD = 20.07) than those with COPD/pulmonary disease (Mean = 72.6 years old, SD = 11.64).

**Table 1.** Patient characteristics by group.

Disease	Without COPD and pulmonary disease	With COPD and pulmonary disease
All	(7741)	(1010)
Age in years	62.5 (SD = 20.07)	72.6 (SD =11.64)
Female	47.0% (3689)	47.4% (479)
Cancer	13.6% (1069)	12.5% (126)
Diabetes (+induced complication)	22.8% (1790)	29.9% (292)
Paraplegia	4.0% (317)	4.6% (46)
Peptic ulcer	0.8% (66)	1.3% (13)
peripheral vascular disease	4.5% (353)	8.2% (83)
Renal disease	16.9% (1325)	20.2% (204)
Acute myocardial infarction	8.7% (685)	17.6% (178)
COPD & pulmonary disease	0.0% (0)	100% (1010)
Connective tissue disorder	3.5% (275)	5.2% (53)
Chronic heart failure	11.9% (938)	25.1% (254)
HIV	0.001% (8)	0.0% (0)
Cerebrovascular accident	2.5% (200)	3.0% (30)
Liver disease	3.0% (235)	2.3% (23)
Dementia	7.2% (567)	8.2% (83)

Following the release of the new oxygen guidelines, oxygen use was applied within SpO_2_ guidelines specifications less than half the time, i.e. 41.6% (*n* = 3638/8751). Overuse was more common 55.0% (4809/8751) than underuse 3.5% (304/8751). Following the release of the dexamethasone guidelines on 25 June 2020, 6397 patients were admitted of which 805 (12.6%) were prescribed dexamethasone. Further analyses for oxygen use are provided below.

### Analysis 1: 7741 patients not experiencing chronic obstructive pulmonary disease/pulmonary disease

For patients not experiencing COPD/pulmonary disease, oxygen was administered within the SpO_2_ guideline recommendations (SpO_2_ levels 92%–96%) for 43.8% (*n* = 3383/7741) of the time; 52.8% (*n* = 4084/7741) were over-administered and 3.5% (274/7741) were under-administered.

Several clinical procedures, pre-admission diagnoses, and demographic factors were associated with adherence (see Table 2). Regarding clinical procedures, as oxygen flow rate increases by a unit, the odds of over-administering were 7% lower, and for under-administering were 15% higher. In addition, patients in the over-prescribed group were 45% more likely to be given dexamethasone. No significant associations were found for dexamethasone.

**Table 2.** Results for patients not experiencing chronic obstructive pulmonary disease/pulmonary disease.

Predictors	Over-administered odds ratio (95% CI) *P*-value	Under-administered odds ratio (95% CI) *P*-value
Clinical procedures		
Oxygen flow rate	0.93 (0.91–0.95) <0.01*	1.15 (1.03–1.18) <0.01*
Dexamethasone	1.45 (1.24–1.69) <0.01*	0.74 (0.58–1.08) 0.15
Number of medications	0.99 (0.99–1.01) 0.95	0.99 (0.98–1.00) 0.38
Pre-admission diagnoses		
Cancer	0.79 (0.68–0.91) <0.01*	1.18 (0.82–1.70) 0.37
Peptic ulcer	0.49 (0.27–0.87) 0.01*	1.28 (0.24–6.88) 0.77
Renal disease	0.79 (0.69–0.91) <0.01*	0.64 (0.49–0.85) 0.01*
Connective tissue disorder	1.15 (0.88–1.48) 0.29	0.37 (0.28–0.49) <0.01*
Chronic heart failure	1.10 (0.93–1.30) 0.23	0.80 (0.58–1.08) 0.14
Paraplegia	0.98 (0.76–1.27) 0.88	1.06 (0.57–1.95) 0.85
Cerebrovascular accident	1.09 (0.79–1.50) 0.60	1.62 (0.67–3.93) 0.29
Peripheral vascular disease	0.89 (0.71–1.12) 0.33	1.60 (0.54–1.39) 0.13
Liver disease	0.80 (0.58–1.11) 0.17	1.19 (0.38–3.76) 0.77
Diabetes	1.05 (0.94–1.18) 0.40	1.03 (0.79–1.34) 0.81
Dementia	1.16 (0.96–1.40) 1.22	1.36 (0.93–2.04) 0.12
Demographics		
Sex	0.94 (0.85–1.04) 0.21	0.90 (0.72–1.14) 0.40
Age	0.98 (0.97–0.98) <0.01*	1.01 (1.00–1.02) 0.02*

^*^Indicates a *P*-value < 0.05.

Regarding diagnoses, patients were more likely to fall in the over-administered group if they were not diagnosed with cancer, peptic ulcer, or renal disease. Patients were more likely to fall in the under-administered group if they were not diagnosed with renal disease or connective tissue disorder. Regarding demographics, female patients were less likely to fall within the under-administered group, and advancing age decreased the likelihood of over-administering and increased the likelihood of under-administering.

### Analysis 2: 1010 patients experiencing chronic obstructive pulmonary disease/pulmonary disease

For patients experiencing COPD/pulmonary disease, oxygen was administered within SpO_2_ guideline recommendations (SpO_2_ levels 88%–92%) for 25.2% (*n* = 255/1010) of patients; 71.8% (725/1010) were over-administered and 3.0% (30/1010) were under-administered.

Several factors were associated with adherence (see Table 3). Regarding clinical procedures, as oxygen flow rate increases by a unit, the odds of over-administering were 11% lower, and for under-administering were 14% higher. No significant associations were found for dexamethasone. Regarding diagnoses, patients were more likely to fall in the over-administered group if they were not diagnosed with renal disease but were diagnosed with chronic heart failure. Patients were more likely to fall in the under-administered group if they were not diagnosed with renal disease or a cerebrovascular accident. Regarding demographics, no significant associations were found for age or sex.

**Table 3.** Results for patients experiencing chronic obstructive pulmonary disease/pulmonary disease.

Predictors	Over-administered odds ratio (95% CI) *P*-value	Under-administered odds ratio (95% CI) *P*-value
Clinical procedures		
Oxygen flow rate	0.89 (0.84–0.96) 0.00*	1.14 (1.00–1.30) 0.04*
Number of medications	1.00 (0.99–1.01) 0.89	1.01 (0.99–1.02) 0.07
Dexamethasone	0.98 (0.54–1.82) 0.98	2.41 (0.28–20.84) 0.42
Pre-admission diagnoses		
Cancer	0.96 (0.61–1.53) 0.88	3.68 (0.47–28.88) 0.22
Peptic ulcer	1.07 (0.28–4.14) 0.93	0.45 (0.04–4.90) 0.52
Renal disease	0.64 (0.42–0.96) 0.03*	0.29 (0.12–0.69) 0.00*
Connective tissue disorder	0.78 (0.40–1.55) 0.48	1.06 (0.13–8.99) 0.96
Chronic heart failure	1.72 (1.21–2.43) <0.01*	1.10 (0.46–2.67) 0.83
Paraplegia	1.16 (0.48–2.81) 0.74	6.19 (0.33–116.90) 0.22
Peripheral vascular disease	1.09 (0.63–1.87) 0.76	0.89 (0.22–3.57) 0.87
Cerebrovascular accident	0.73 (0.24–2.24) 0.59	0.09 (0.01–0.99) 0.04*
Liver disease	0.69 (0.24–1.94) 0.48	^a^
Diabetes	1.23 (0.88–1.71) 0.23	0.96 (0.42–2.22) 0.93
Dementia	0.98 (0.57–1.69) 0.95	1.21 (0.25–5.85) 0.82
Demographics		
Age	1.00 (0.99–1.02) 0.81	0.99 (0.95–1.02) 0.56
Sex	0.34 (0.86–1.56) 0.34	1.22 (0.55–2.71) 0.63

a This parameter is set to zero because it is redundant (SPSS cancel command variable).

^*^Indicates a *P*-value < 0.05.

## Discussion

### Statement of principal findings

Suboptimal use of oxygen was common during the first wave of the COVID-19 pandemic within the Trust. Oxygen use was applied within stated guidelines less than half the time, i.e. 41.6%. In terms of absolute percentages, under-administering was low across groups (<6%), and over-administering was far more common for patients diagnosed with COPD/pulmonary disease (71.8%) than for those not (52.8%). In addition, dexamethasone was used in only 12.6% of cases recommended by guidelines. Simply issuing new guidelines for oxygen therapy and dexamethasone was not sufficient to realize their implementation.

### Strengths and limitations

Strengths of the present study include the unique time in which it was tested, i.e. during the COVID-19 pandemic when many new guidelines were disseminated, and the large number of patients included. Several limitations of the study should be noted. Firstly, our observational method permits comments about associations between factors and oxygen use but not about causal relationships between factors. Secondly, as the sample sizes for our two regression analyses are different, the results for patients not diagnosed without COPD/pulmonary disease are more precise than for those diagnosed with COPD/pulmonary disease. Thirdly, the pre-admission diagnoses cannot capture undiagnosed disease states. Lastly, nearly 10% of unique patient records (*N* = 899/9650 in Fig. 1) were excluded due to missing records, and only the first instance of oxygen therapy recorded for each patient was included in our analyses; subsequent treatments (the 5537 multiple records in Fig. 1) may have been within guidelines specifications. Still, the present findings for oxygen overuse align well with previous multi-country quantitative reviews conducted before COVID-19 [15]. While randomized controlled trials found that oxygen (compared with air) does not relieve breathlessness in the absence of severe hypoxemia [16], patients and health workers continue to perceive oxygen as a life-sustaining force [17, 18].

### Interpretation within the context of the wider literature

The current study focused on guidelines adherence for oxygen therapy in terms of target SpO_2_ levels. In 2019, JAMA published a structured review of 25 studies describing the negative effects of more liberal use of oxygen therapy (outside SpO_2_ guidelines) compared to more conservative use [3]. Not all studies agree, and differences in patient outcomes may be due to patient-specific factors. In particular, patients with COPD/pulmonary disease are more negatively influenced by higher saturation levels than those without, and current guidelines advise lower targets for such patients in England. The existence of different guidelines for subgroups renders guideline compliance more challenging, and, in the current study, over-administering was far more common for COPD/pulmonary patients. A 2022 trial [19] conducted in the United States randomized 2541 critically ill patients to receive lower (88% to 92%) intermediate (92% to 96%) or higher target saturations (96% to 100%). They found no difference in patient outcomes. Where patient outcomes do not differ, lower target levels for all patients could be advised, which would protect patients diagnosed with COPD/pulmonary disease and make guideline adherence easier. That said, the current paper does not investigate patient outcomes, and any changes to guidelines should consider subgroups that might be disadvantaged by such changes.

While qualitative studies with health workers and caretakers can reveal negative attributes of oxygen therapy, e.g. difficulty administering oxygen, themes largely drift towards positive attributions of oxygen therapy, e.g. ‘oxygen giveth’ and ‘oxygen as a panacea’ [20, 21]. As Pilcher and Beasley stated in 2015 ‘A major change is needed in the entrenched culture of routinely administering high-concentration oxygen to acutely ill patients regardless of need’. [22] This call for change was not heeded during the first wave of the COVID-19 pandemic.

Simply issuing guidelines can be insufficient to change clinical practice [23]. Implementation of guidelines in clinical practice involves a confluence of factors across the individuals, the systems in which they work, and the people they are meant to serve [24]. One factor complicating oxygen therapy is that the recommended saturation rate changes depending on previous diagnoses, i.e. COPD/pulmonary disease. While educational interventions might help health workers rationally decide what optimal care could look like in advance, the life-saving potential of oxygen use that they may have personally witnessed may render those guidelines difficult to implement when a patient appears breathless [25]. Here, continuing education alone is unlikely to help. Such education could be complemented with additional support.

### Implications for policy, practice, and research

Additional support could be informed by a thorough behavioural assessment of the barriers and facilitators health workers experience to applying oxygen therapy within guidelines [26]. For instance, Rose *et al*.’s [27] project, which ultimately improved oxygen prescribing, started with a behavioural assessment in which a fishbone diagram containing 16 potential root causes was collaboratively constructed. Some root causes likely require social interventions, e.g. personalized emails or texts from trusted messengers or champion roles similar to those used to promote hand hygiene and optimal antibiotic prescribing [28]. Barriers related to memory might be overcome with reminder prompts, e.g. stickers placed near the oxygen dispensing equipment [29], or prompts inserted into an electric prescribing software. However, rather than focusing on helping healthcare workers use oxygen therapy, future interventions could focus on helping healthcare workers to stop using oxygen therapy where it is not needed or where sufficient oxygen levels are already achieved. Role-playing (i.e. simulating) what it is like to choose not to continue applying oxygen therapy may prove a useful addition to education. Here, people can practice choosing to not apply oxygen or to cease oxygen therapy sooner. They could also practice supporting co-workers who make these choices. Additionally, framing watchful waiting as an active treatment choice may help health workers to make this decision more confidently [30].

## Conclusions

The current study focused on adherence with oxygen guidelines in England. Similar to previous studies, we find that overuse is more common than underuse. This finding was true for both newly issued guidelines (for non-COPD patients) and long-existing guidelines (for COPD patients). Interventions to optimize oxygen use will likely require more support than rational guidelines and skills training typically provide. Future interventions could also tap into other influences on oxygen use (e.g. social support, memory, and emotions) not only to help workers choose to apply oxygen therapy but also to help them choose to not apply or to cease administering oxygen therapy before recommended thresholds are surpassed.

## Data sharing statement

The data underlying this article were provided by University Hospital Birmingham NHS Foundation Trust by permission. Data will be shared on request to the corresponding author with the permission of the University Hospital Birmingham NHS Foundation Trust.
